# Parliament and the Professions

**Published:** 1921-04-02

**Authors:** 


### Parliament and the Professions.

### Industrial Fatigue.

Dr. Addison, in answer to a question, stated that the work of the Industrial Fatigue Research Board, upon which both the Home Office and the Ministry of Labour are represented, is being maintained by the Medical Research Council under a new scheme of organisation, and the Board are already engaged in an extensive inquiry into the cotton trade, the result of which will, it is expected, be applicable to a large extent to all textile trades. When the report of this investigation has been received it will be considered how far inquiry can profitably be extended to other textile industries, including the woollen trade.

### Venereal Disease.

Captain Elliot inquired of the Minister of Health as to Government subsidy of the National Council for Combating Venereal Disease, and Dr. Addison replied that in accordance with the recommendation of the Royal Commission the National Council has been recognised since 1916 as the authoritative body for the purpose of spreading knowledge and giving advice in regard to the question of venereal diseases in its varied aspects. During the years ending March 31, 1919, and March 31, 1920, grants of £13,000 and £15,600 respectively had been made, and the grant to date for the current financial year amounts to £13,750. These sums have been paid in respect of the expense of general publicity.

### Cost of Health Services.

Replying to questions, Dr. Addison stated that the rate of infant mortality in London has fallen during the last four years as follows: 1917, 104; 1918, 108; 1919, 85; 1920, 75. The average cost to the rates of London of the maternity and child-welfare service was during each of the last four years considerably less than one penny per pound of assessable value. The grants paid to local authorities in London in respect of tuberculosis, maternity welfare, and venereal disease during the last completed financial year were: Tuberculosis, £49,195; maternity and child-welfare, £39,763; venereal disease, £35,543; total, £124,501. The total expenditure of all these services combined out of an average rate in London of 14s. 5d. for the year 1920-21 was 2½d.

### Nurses' Co-operation.

Mr. Maclean asked the President of the Board of Trade whether the Nurses' Co-operation, 22 Langham Street, is covered by Articles of Association approved by him; if so, whether he is aware that seven out of nine nurses who were members of the Committee of Management have been thrown off the Committee because they have made complaints with regard to the management, especially in respect of large sums of money paid out without their knowledge and consent; that three of these ladies, who were elected by their colleagues to the Committee of Management to safeguard the interests of nurses, have been expelled from the organisation and deprived of their living because they asked for a meeting to be convened to hear their complaints; and whether he will make inquiry into the matter.

Sir P. Lloyd-Greame replied that the Board of Trade in 1894 issued a Licence under Section 23 of the Companies Act, 1867 (now Section 20 of the Companies (Consolidation) Act, 1908), to the Nurses' Co-operation, and approved the form of the Memorandum and Articles of Association. A letter has recently been received from one of the nurses with regard to the matters referred to in the question, and the statements made in that letter are being considered.

